# Effects of Melatonin and Silymarin on Reactive Oxygen Species, Nitric Oxide Production, and Sperm Viability and Motility during Sperm Freezing in Pigs

**DOI:** 10.3390/ani13101705

**Published:** 2023-05-21

**Authors:** Sang-Hee Lee, Seunghyung Lee

**Affiliations:** College of Animal Life Sciences, Kangwon National University, Chuncheon 24341, Republic of Korea

**Keywords:** boar sperm, melatonin, silymarin, freezing, semen

## Abstract

**Simple Summary:**

Sperm has oxidative stress during freezing in boar semen. Oxidative stress causes reactive oxygen species to occur and produces and nitric oxide. The viability and motility of sperm are damaged by oxidative stress. We investigated the effects of melatonin and silymarin on reactive oxygen species, nitric oxide production, sperm viability, and motility in frozen–thawed boar semen. As a result, melatonin and silymarin elevated the viability and motility of sperm, and also reactive oxygen species and nitric oxide production decreased. Therefore, we suggested that melatonin and silymarin are beneficial for antioxidants during freezing sperm in pigs.

**Abstract:**

Sperm during the freezing and thawing process is damaged by oxidative stress. Thus, its antioxidant scavenger is essential for sperm survival and death in frozen–thawed semen. We used melatonin and silymarin in experiments after the dose-dependent experiment. Our study aimed to identify the effect of melatonin and silymarin on the motility and viability of sperm, reactive oxygen species (ROS), and nitric oxide (NO) production in frozen–thawed boar semen. Melatonin and silymarin were treated alone and cotreated in the fresh boar semen. Boar semen was collected using the gloved-hand method from ten crossbred pigs, and samples were used in the experiments. We evaluated sperm viability using SYBR-14 and PI kit, and ROS and NO production were detected by DCF-DA and DAF-2, respectively. The sperm motility was not significantly different between non-treatment and treatment. ROS and NO production in frozen–thawed sperm were decreased by melatonin and silymarin. Moreover, silymarin significantly reduced NO production more than melatonin. Melatonin and silymarin enhanced the viability of sperm. We suggest that melatonin and silymarin are essential antioxidants in semen cryopreservation for protecting sperm damage and maintaining sperm viability. Melatonin and silymarin may be useful antioxidants in freezing boar sperm.

## 1. Introduction

Oxidative stress is a phenomenon that occurs when there is an imbalance between the production of reactive oxygen species and the antioxidant defense mechanisms. Oxidative stress can lead to the damage of cells and tissues, including in the male reproductive system [1,2]. Sperm are particularly vulnerable to oxidative stress due to their high levels of unsaturated fatty acids in the membranes and their limited antioxidant capacity. Oxidative stress can also affect the function and fertility of sperm, DNA, and protein damage [1,3,4]. Moreover, oxidative stress is a well-recognized consequence of the process of freezing sperm for reproductive techniques. During the freezing and thawing process, sperm is exposed to a range of environmental stresses, including fluctuations in temperature and exposure to cryoprotectants [4]. The stress can lead to the production of reactive oxygen species, which can cause damage to sperm cells by oxidizing lipids, proteins, and DNA. The oxidative damage can also lead to reduced sperm motility, viability, and fertility [4,5]. In addition, ROS-induced DNA damage can result in chromosomal abnormalities and impaired embryo development, which can compromise the success of assisted reproductive techniques, such as in vitro fertilization and intracytoplasmic sperm injection [6]. Thus, understanding the mechanisms of oxidative stress in freezing sperm and identifying effective antioxidants are important fields of research, with potential implications for improving the success of assisted reproductive techniques and the outcomes of fertility treatments. 

Since organelles in sperm disappear during spermatogenesis, the ability to remove reactive oxygen species (ROS) and nitric oxide (NO) generated from the frozen sperm cells is significantly lower than that of normal somatic cells [7,8]. In fact, ROS and NO are important molecules that play a critical role in the process of freezing sperm [9]. ROS is a type of molecule that contains oxygen and has the potential to cause damage to sperm cells. On the other hand, NO is a gas that is involved in a wide range of physiological processes, including spermatogenesis [10,11]. Moreover, the oxidative stress by ROS and NO that happens during the freezing and thawing process to preserve sperm is the cause of significantly reduced sperm viability [12]. Therefore, scavenging of ROS and NO is important in freezing sperm, since cryopreservation can lead to an increase in ROS and NO levels, which can cause oxidative stress and damage to sperm cells. Moreover, scavengers of ROS and NO, such as antioxidants and NO regulators, can help to reduce oxidative stress and prevent damage to sperm and to sperm cells during freezing and thawing, thereby improving the quality and viability of the sperm.

Generally, non-permeable cryoprotectants (alpha-lactose and egg yolk) and permeable cryoprotectants (glycerol and Orvus Es Paste, OEP) are used to freeze boar sperm [13]. The reason for using the freeze protection agent is to prevent damage to the sperm and to protect the sperm to maintain its function of the sperm. Although the release of intracellular water by the cryoprotectant is essential in the sperm freezing process, the sperm undergoes osmotic stress during this process [13,14]. As a result, it has been reported that ROS generates in sperm cells, and the sperm are damaged. For this reason, it is critical to maintain the viability of sperm with an appropriate concentration and cotreatments of antioxidants and to prevent sperm loss from oxidative stress.

ROS, which occurs in the freezing and thawing process of semen, is a typical cause of oxidative stress. An increase in ROS causes a decrease in sperm motility, viability, and fertilization ability [15,16]. In addition, since NO induces apoptosis and rapidly decreases the viability of sperm, suppressing or reducing the generation of NO is essential for protecting the frozen sperm [17,18]. Based on previous studies, we identified the concentrations of ROS and NO generated during the freezing–thawing process of semen. We treated melatonin and silymarin to increase the viability of sperm. Generally, melatonin and silymarin are antioxidants. Melatonin is the scavenger of ROS and free radicals in animals and humans [19]. In the brain, Melatonin is secreted by the pineal gland and has an essential function in the neuroendocrine system [20]. Moreover, melatonin regulates sperm quality and male reproduction, but it is not clear in the reproductive system of males, such as sperm in freezing semen. In particular, silymarin is a drug used to treat liver disease, and its action is known to protect and detoxify liver cells by removing ROS [21]. However, there are no studies on the effects of ROS and NO, which generate during the freezing–thawing process of boar semen, with melatonin and silymarin in frozen–thawed sperm.

The process of freezing sperm for assisted reproductive techniques can result in oxidative stress, which can lead to damage to the sperm’s DNA and reduced fertility, sperm motility, and viability [22,23]. Thus, there has been interest in identifying natural antioxidants that can help protect frozen sperm from oxidative damage and preserve sperm quality. Melatonin is a hormone that regulates the sleep–wake cycle and has been shown to have potent antioxidant properties [24]. It has been suggested that melatonin may help protect sperm from oxidative stress during the freezing process. Some studies have found that melatonin supplementation can improve sperm motility and increase the success rate of assisted reproductive techniques using frozen sperm [25,26]. Silymarin, a flavonoid extracted from milk thistle, has also been investigated for its potential protective effects on frozen sperm [27,28]. As melatonin, silymarin has been shown to have antioxidant properties, and research has suggested that it may help prevent oxidative damage to sperm membranes and improve sperm motility. Nevertheless, these substances represent an intriguing avenue for potentially improving the success of assisted reproductive techniques using frozen sperm. However, the physiological mechanism has not yet been clearly identified.

This study investigated how melatonin and silymarin act as antioxidants in sperm from boar frozen–thawed semen, which have not been identified yet. Moreover, we investigated the effect of melatonin and silymarin treatment on the viability of sperm. Since research on the effect of melatonin and silymarin on frozen–thawed semen is unclear, it is necessary to investigate which part of melatonin and silymarin plays a role in ROS and NO to affect sperm during the freezing and thawing of semen. Thus, this study aimed to identify the effect of melatonin and silymarin on frozen–thawed sperm. We examined the motility and viability of sperm, ROS, and NO production in frozen–thawed boar semen with melatonin and silymarin.

## 2. Materials and Methods

### 2.1. Animal

We used ten crossbred pigs (Duroc × Yorkshire × Landrace, average ages = 28.7 ± 3.2 months) in an artificial insemination center (Wonju, Republic of Korea). All experiments and guidelines were followed by the Institutional Animal Care and Use Committee of the University (KIACUC-09-0139).

### 2.2. Chemicals

Melatonin and silymarin were purchased from Sigma-Aldrich (St. Louis, MO, USA). Unless otherwise indicated, all reagents used in this study were purchased from Sigma-Aldrich (St. Louis, MO, USA).

### 2.3. Preparation of Semen

Fresh semen from pigs was collected by a glove-hand method. Collected semen was diluted in a long-term swine liquid semen extender (Modena SGI, Table 1), transported to the laboratory, and then stored at 18.0 °C before the freezing experiment. Fresh semen samples were subjected to microscopic analysis selecting semen samples with at least 80% motile sperm [29].

### 2.4. Freezing and Thawing of Semen

First, the semen samples were centrifuged to remove seminal plasma for removing seminal plasma at 400× *g* for 5 min, and then, sperm fraction was only used for cryopreservation [30,31]. Briefly, the first freezing extender was composed of 11.0% α-lactose (Sigma, St. Louis, MO, USA) and 20.0% egg yolk, and second freezing extender was made from the first freezing extender supplemented with 9.0% glycerol (Sigma) and 1.5% OEP (Nova Chemical Sales Inc., Scituate, MA, USA). We added antioxidants in a freezing extender, such as 0.0, 0.1 mM melatonin (Sigma), 0.01 mM silymarin (Sigma), and mixed melatonin and silymarin. Sperm were diluted with the first freezing extender at 18 °C and was cooling at 5 °C for 120 min, then diluted with the second freezing extender at 5 °C of the half volume of the first freezing extender until 1.0 × 10^9^ sperm/mL. Sperm was packaged into 0.5 mL straw (20 straws/semen/pig) and cooled to −120 °C for 10 min before being plunged into liquid nitrogen for storage using static nitrogen vapor [29,30]. The frozen sperm into straw was thawed in a water bath at 37.0 °C for 45 s and centrifuged without Modena at 400× *g* for 5 min and carefully removed the freezing extender. Then frozen sperm were washed with Modena two times at 410 g for 5 min [30,31]. After washing, the samples were resuspended to Modena until 1.0 × 10^7^ sperm/mL for analysis of sperm viability, ROS, and NO production.

### 2.5. Evaluation of Sperm Motility

The motility of sperm was subjectively evaluated according to a standard method. To measure fresh and frozen–thawed sperm motility, a 10.0 μL sperm sample was placed on a pre-warmed slide glass and covered with a cover glass and then put in the sample on a warm chamber. The total sperm motility was subjectively assessed by visual estimations. In at least five fields, sperm motility was determined and examined at ×200 magnification under a light microscope (Olympus, Tokyo, Japan).

### 2.6. Sperm Viability Assay

The viability of frozen–thawed sperm was evaluated using a sperm Live/Dead kit (L-7011, Invitrogen, New York, NY, USA) according to a previous study [31]. The sperm samples were diluted with 40 nM SYBR-14 and 2.0 μM propidium iodide (PI), which were incubated at 38 °C for 5 min in a dark room, and then these samples were centrifuged at 410× *g* for 5 min. After the supernatant was carefully removed, the sample was resuspended in Phosphate Buffered Saline (PBS). Then, sperm were measured using flow cytometry (488nm, FACSCaliber, BD Biosciences, Franklin Lakes, NJ, USA). Dot plot analysis conditions regarding forward scatter and side scatter, FL-1, and FL-2 were set according to a previous study [31]. The dot plot data were analyzed using CELLQuest software (Version 6.0 Becton Dickinson, San Jose, CA, USA, and dot plots of SYBR-14 positive and PI negative were considered viable sperm.

### 2.7. Reactive Oxygen Species Determination

In the thawed sperm, we measured the production of reactive oxygen species (ROS) using a 2′,7′-dichloro-fluorescein diacetate (DCF-DA, Invitrogen) [32]. The produced ROS was quantified by the DCF standard curve. After centrifuging a 1.0 mL semen sample with a concentration of 1.0 × 10^7^ sperm/mL at 410× *g* for 5 min, 200.0 μL of the supernatants was mixed with 20.0 μL of 20.0 mM DCF-DA and incubated for 30 min in an incubator at 37 °C, and then these samples were centrifuged at 400× *g* for 5 min. After the supernatant was carefully removed, the sample was resuspended in Phosphate Buffered Saline (PBS). Then, sperm were measured using flow cytometry (BD Biosciences). The ROS production was analyzed using a histogram (CELLQuest software, Becton Dickinson) and ROS production was normalized using the control treatment values.

### 2.8. Production of Nitric Oxide

The production of nitric oxide was detected using a nitric oxide 4,5-diaminofluorescein diacetate (DAF-2 DA) reagent (Sigma) [26,33]. Briefly, we used a concentration of 1.0 × 10^7^ sperm/mL in PBS at 410 g for washing for 10 min, and then DAF-2 DA was added to the suspension for a final concentration of 10.0 μM. Finally, the sample incubated at 37 °C for 1 h in a dark room. These samples were centrifuged at 400× *g* for 5 min. After that, we measured the incubated samples for NO production of sperm using flow cytometry [18].

### 2.9. Statistical Analysis

One-way ANOVA followed by Fisher-protected least significant difference (PLSD) analysis was used for all statistical data analysis, using Stat View (SAS Institute, Cary, NC, USA). Data were presented as mean ± standard error mean. A *p*-value < 0.05 probability was considered significant.

## 3. Results

### 3.1. Effects of Melatonin and Silymarin on Sperm Motility

**Figure 1:**
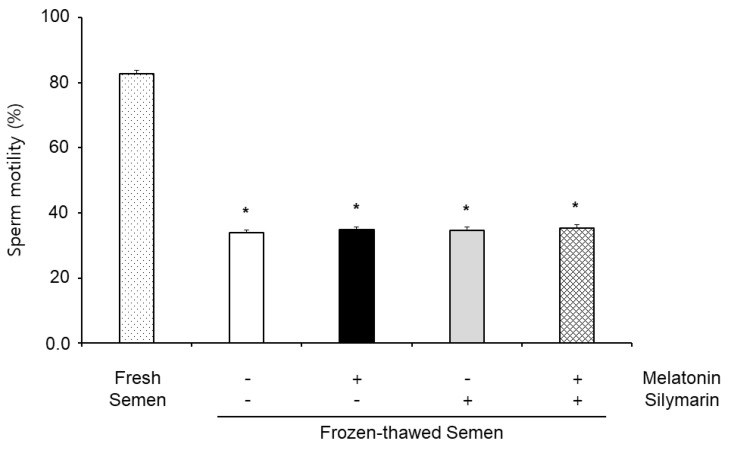
Effect of melatonin and silymarin on sperm motility in the frozen–thawed boar semen and fresh semen. Sperm was treated with 0.1 mM melatonin and 0.01 mM silymarin. Bars represent means ± standard error (SE). Asterisks are significantly different (*, *p* < 0.05).

The effects of melatonin and silymarin on sperm motility are shown in Figure 1. Sperm motility in frozen–thawed semen (33.82%) was significantly decreased compared with fresh semen (82.78%), but the sperm motility was not significantly different between non-treatment (33.82%), 0.1 mM melatonin (34.74%) and 0.01 mM silymarin (34.60%) treatments, and both treatments (35.34%). There was no difference in the treatment of melatonin and silymarin in frozen–thawed semen.

### 3.2. Dose-Dependent Effects of Melatonin and Silymarin on Sperm Viability

We first evaluated the viability of sperm on melatonin and silymarin concentrations in frozen–thawed semen.

Table 2 shows that sperm viability on the frozen–thawed semen was significantly increased in dose-dependent 0.1 mM melatonin (45.52 ± 1.07%) compared to 0 mM (41.71 ± 0.84%) and 0.01 mM (42.72 ± 0.67%) (*, *p* < 0.05). However, sperm viability was decreased at 1.0 mM melatonin treatment compared to the control. In addition, 0.01 mM silymarin (45.86 ± 1.24%) significantly improved viability compared to 0 mM (39.31 ± 0.60%) and 0.001 mM (41.33 ± 1.04%) (*, *p* < 0.05, Table 3). Therefore, we used a concentration of 0.1 mM melatonin and 0.01 mM silymarin for determining ROS, NO production, and viability of sperm in frozen–thawed semen.

### 3.3. Effects of Melatonin and Silymarin on ROS and NO Production and Sperm Viability

**Figure 2:**
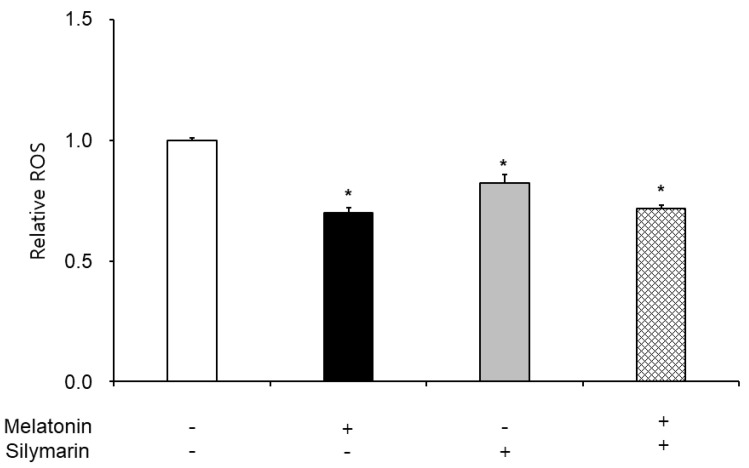
Effect of melatonin and silymarin on ROS in the frozen–thawed boar semen. Sperm was treated with 0.1 mM melatonin and 0.01 mM silymarin. Bars represent means ± standard error (SE). Asterisks are significantly different (*, *p* < 0.05).

**Figure 3:**
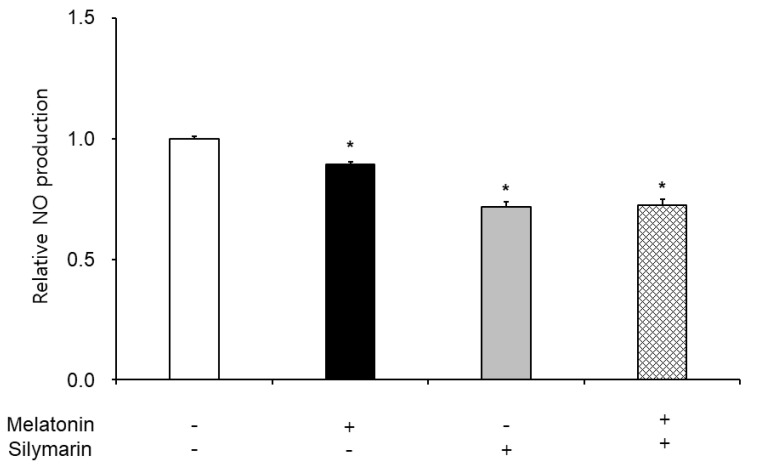
Effect of melatonin and silymarin on NO production in the frozen–thawed boar semen. Sperm was treated with 0.1 mM melatonin and 0.01 mM silymarin. Bars represent means ± standard error (SE). Asterisks are significantly different (*, *p* < 0.05).

**Figure 4:**
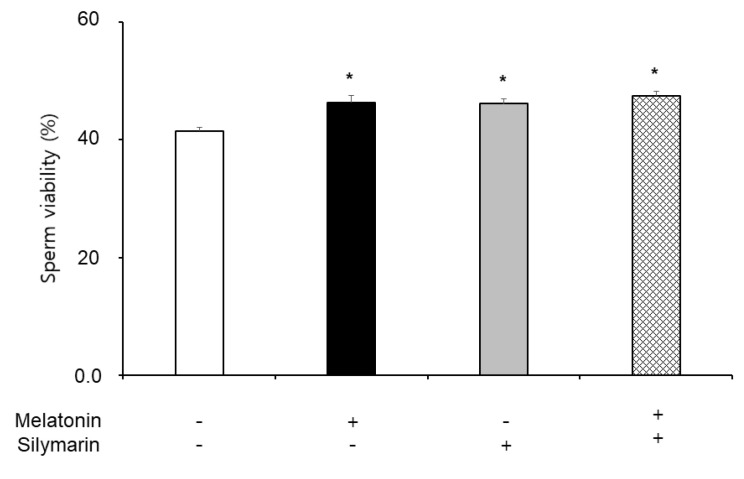
Effect of melatonin and silymarin on sperm viability in the frozen–thawed boar semen. Sperm was treated with 0.1 mM melatonin and 0.01 mM silymarin. Bars represent means ± standard error (SE). Asterisks are significantly different (*, *p* < 0.05).

The effects of melatonin and silymarin on the ROS and NO production are shown in Figure 2 and Figure 3. ROS and NO production in the melatonin and silymarin treatment groups were significantly decreased (*, *p* < 0.05), and both treated groups were significantly lower than the non-treated group and the not-alone-treated groups (*, *p* < 0.05). Finally, the effects of melatonin and silymarin on the viability of sperm are shown in the Figure 4. Sperm viability in both melatonin and silymarin-treated groups was higher than in the non-treated group, but not significantly different between the alone-treated groups (*, *p* < 0.05).

## 4. Discussion

The current study shows that sperm motility did not observe any significant differences in the frozen–thawed semen. Although melatonin and silymarin did not affect sperm motility, frozen–thawed sperm had damage to sperm viability. It was suggested that melatonin and silymarin are used for recovery and enhanced viability in freezing boar semen.

In the present study, the treatment of melatonin and silymarin elevated the viability of frozen–thawed sperm, as was also the case with the cotreatment. Melatonin supplementation in boar semen during liquid storage at 17 °C improved sperm viability, motility, and DNA integrity [24]. Melatonin supplementation reduced the levels of ROS and lipid peroxidation in sperm, indicating that it has potent antioxidant properties. Yoon et al. demonstrated that melatonin maintains mitochondrial membrane potential and decreases excessive intracellular ROS levels in boar sperm [25]. Melatonin treatment improved sperm motility, viability, and membrane integrity during liquid storage at 7 °C. The study also suggested that melatonin can protect sperm from oxidative stress by regulating mitochondrial function. Succu et al. reported that melatonin protects ram spermatozoa in cold shock [34], and the reduction in melatonin inducing oxidative damage in humans has been demonstrated [35]. Karimfar et al. suggested that melatonin exerts its cryoprotective effects on spermatozoa, possibly by counteracting intracellular ROS, such as increasing the motility and viability of sperm in humans [19]. Overall, reports agree that melatonin enhances the viability of sperm from frozen–thawed semen in animals and humans. Moreover, melatonin supplementation during cryopreservation of boar sperm improves sperm motility, viability, membrane integrity, and acrosome integrity. Melatonin reduces the level of ROS and lipid peroxidation, and increases the activity of antioxidant enzymes, such as catalase and glutathione peroxidase. These results suggest that melatonin can protect boar sperm from oxidative stress-induced damage during cryopreservation. Melatonin may have potential applications in improving the fertility of boars in pig production by enhancing the quality of cryopreserved semen.

Silymarin also increased sperm viability in the boar sperm [27,28]. Silymarin is a natural antioxidant compound that has been shown to have a protective effect on boar sperm during cryopreservation, which involves freezing and thawing semen. Wang et al. and Tuncer et al. groups reported that silymarin supplementation during cryopreservation of boar sperm improves sperm motility, viability, and membrane integrity [27,28]. Silymarin also reduced the level of ROS and lipid peroxidation, and it increased the activity of antioxidant enzymes, such as superoxide dismutase and catalase. They also demonstrated that silymarin-treated sperm had a higher fertilization rate, fertility rate after artificial insemination, and embryo development rate in vitro compared to untreated sperm. Moreover, Tuncer et al. showed that silymarin supplementation during cryopreservation of boar sperm reduced the level of ROS and DNA damage, resulting in improved sperm quality. Silymarin also increased the activity of antioxidant enzymes, such as glutathione peroxidase and catalase, and decreased lipid peroxidation. Overall, these studies suggest that silymarin can protect boar sperm from oxidative stress-induced damage during cryopreservation. Silymarin may also have potential applications in improving the fertility of boars in pig production by enhancing the quality of cryopreserved boar semen and others. In addition, some research on silymarin is about hepatotoxic diseases, tumors, and carcinogenesis [36,37,38]. Recently, Etemadi et al. reported that silymarin regulates cadmium-induced apoptosis in human spermatozoa [39]. Cadmium increases ROS and lipid peroxidation levels in cells and rat testes [40]. To our results, silymarin decreased ROS and NO production in frozen–thawed boar sperm. It may be a scavenger for ROS and NO in the freezing and thawing process of sperm. ROS and NO mediate apoptosis through oxidative stress in the cells. Cryopreservation is also essential for freezing sperm because it protects sperm osmotic pressure and oxidative stress in the freezing semen. Thus, we strongly suggest that silymarin is a potential antioxidant in frozen–thawed semen.

In this study, we have deeply discussed the effect of silymarin on sperm in frozen–thawed semen because many research groups reported that melatonin has the function of an antioxidant in the cells and improves the preservation of sperm function and quality. Nevertheless, the viability of sperm in freezing semen is lower than in fresh semen in humans and animals. Oxidative stress, such as free radicals, reactive oxygen species, and nitric oxide, leads to cell death. Thus, the viability of sperm in frozen–thawed semen is lower than in fresh semen. Especially, Ferrusola et al. suggested that cryopreservation induces nitric oxide production in spermatozoa [19]. In the results of this study, we found that the antioxidant effects of melatonin and silymarin were different, carefully. Melatonin was involved in the general inhibition of ROS, and silymarin was involved in the inhibition of nitrogen oxide. There was a possibility that the treatment of silymarin inhibited nitric oxide synthesis. Thus, we suggest that silymarin treatment could inhibit nitric oxide synthesis.

Although the antioxidant function of silymarin is known, there is no study on which mechanism has an antioxidant effect. Some other studies reported that silymarin regulates the spermatogenesis process in rats [41]. In bovine oviduct epithelial cells, Jang et al. reported that silymarin affects the survival rate in NO-induced oxidative stress experiments [42]. NO is synthesized via neuronal NOS (nNOS, NOS1), inducible (iNOS, NOS2), and endothelial NOS (eNOS, NOS3). Especially, NOS1 and NOS2 are soluble and found in the cytosol, and NOS3 is membrane-associated [43,44]. ROS enhanced NO production in macrophages with iNOS expression, but LPS-stimulated ROS was decreased by an iNOS inhibitor [45]. Salerno et al. reported that antioxidant inhibits nitric oxide synthase, nNOS, and eNOS [46]. Since NO production is normally controlled by NOS (nNOS, iNOS, and eNOS), it is important in cells and also in the sperm. We suggest that NO production regulates scavenging free radicals or inhibiting nitric oxide synthase in the frozen–thawed sperm. Thus, we are testing the nitric oxide synthesis experiment with silymarin to identify nitric oxide production in frozen–thawed sperm. Therefore, based on the results, we are experimenting to find out how nitric oxide synthesis by silymarin occurs. These results will be useful for understanding the novel function and mechanism of silymarin in boar semen.

## 5. Conclusions

In conclusion, this study on the role of melatonin and silymarin in ROS, NO production, sperm motility, and viability showed that melatonin and silymarin are regulated in frozen–thawed boar semen. Moreover, both melatonin and silymarin may play a role in preserving the quality of frozen sperm. These results identified melatonin and silymarin-enhanced sperm viability and decreased ROS and NO production in the frozen–thawed boar semen. These results suggest that melatonin and silymarin can be used as the main antioxidant during sperm freezing in pigs. Furthermore, silymarin probably regulates nitric oxide synthesis more than ROS in freezing boar sperm. Thus, we need to study nitric oxide synthesis with silymarin to understand the specific function in boar semen. Moreover, regulated studies are needed to fully understand the mechanisms by which they work and to determine the optimal dosages and administration protocols for their use in preserving frozen sperm. Overall, it seems that melatonin and silymarin both have the potential as protective agents for frozen sperm, but more research is needed to fully establish their efficacy and safety.

## Figures and Tables

**Table 1:** Composition of Modena extender.

	Ingredients	Company	Cat. #	Concentration (g/L)
Modena ^1^	Glucose	Sigma	G8270	25.0
	EDTA		E5134	2.25
	Sodium citrate		S4641	6.90
	Sodium bicarbonate		S5761	1.00
	Tris		T6066	5.65
	Citrate		C1909	2.00
	Cysteine		7880	0.05
	BSA ^2^		A4503	3.00
	Gentamicin sulfate	Sigma	G3632	0.30
	pH	-	-	7.00

^1^ Modena extender. ^2^ Did not add BSA for storage of liquid semen for the long-term extender.

**Table 2:** Dose-dependent effect of melatonin on the viability of sperm in frozen–thawed boar semen.

	Melatonin (mM)			
Viability	0	0.01	0.1	1.0
	41.71 ± 0.84	42.72 ± 0.67	45.52 ± 1.07 *	39.17 ± 0.45

* Values in the same low with different superscripts are significantly different (*, *p* < 0.05), mean ± SEM.

**Table 3:** Dose-dependent effect of silymarin on the viability of sperm in frozen–thawed boar semen.

	Silymarin (mM)			
Viability	0	0.001	0.01	0.1
	39.31 ± 0.60	41.33 ± 1.04	45.86 ± 1.24 *	37.75 ± 1.85

* Values in the same low with different superscripts are significantly different (*, *p* < 0.05), mean ± SEM.

## Data Availability

No new data were created or analyzed in this study. Data sharing is not applicable to this article.
